# New findings on palynofacies characteristics of semi-enclosed deep-sea environments in the East Sea over 2 million years

**DOI:** 10.1038/s41598-020-73493-3

**Published:** 2020-10-02

**Authors:** Yongmi Kim, Sangheon Yi, Chang-Pyo Jun, Eunmi Lee, Gil Young Kim

**Affiliations:** 1 Korea University of Science and Technology, Daejeon, 34113 South Korea; 2 Korea Institute of Geoscience and Mineral Resources, Daejeon, 34132 South Korea; 3 Kangwon National University, Chuncheon, 24341 South Korea

**Keywords:** Climate sciences, Ocean sciences

## Abstract

Phytoclasts in the form of plant debris in terrestrial sediments can be transported by water to distant areas because they are lighter than inorganic particles. The semi-enclosed East Sea, which is connected by narrow straits to other seas, is adjacent to continental shelves that are the source area of terrestrial sediment flowing into the East Sea. These shelves alternated repeatedly between terrestrial and marine environments as a result of eustatic sea-level changes during the Late Quaternary. Palynofacies analyses of the IODP Exp. 346 U1430 core, located in the Eastern South Korea Plateau (ESKP) of the East Sea, have revealed changes in the size and concentration of phytoclasts associated with glacial–interglacial cycles. These changes are generally negatively correlated with the global sea-level curve, and their anti-phase cycles with high amplitude are clearly evident during the last ca. 750 ka with the geotectonic stabilization period. In particular, several coarse-grained phytoclasts were observed during the glacial period, including the Last Glacial Maximum (LGM). These findings suggest that the concentration and size of phytoclasts flowing into the East Sea were influenced by changes in the distance of the source area, depending on the water depth of the strait and nearby shelves owing to sea-level changes in tandem with glacial–interglacial cycles and geotectonic events.

## Introduction

Phytoclasts are plant-derived organic matter comprising clay- to fine sand-sized particles, excluding palynomorphs and amorphous (structureless) organic materials. Phytoclasts may include black-brown wood fragments, leaf-derived cuticles, plant tissue, and charcoal; woody particles containing acid-resistant lignin are particularly easily preserved under any condition^1,2^. Generally, in dense, closed forest (e.g., in eastern China and on the Korean Peninsula), some of the anemophilous pollen produced in the canopy can be moved above the canopy by air currents and transported by monsoons. Most phytoclasts and pollen from understory shrubs and herbs are carried by air movement, deposited on the forest surface, and subsequently transported by water^3^. Occasionally, fine-grained phytoclasts in regions with open vegetation, such as the highland desert, are transported by the winter monsoon (e.g., aeolian dust). However, the concentration of wind-driven phytoclasts from open vegetation is low because such areas are generally covered by herbs and shrubs, rather than by trees. Unlike coniferous pollen transported by wind, most woody particles are delivered and deposited like herb pollen and transported via water along with other terrestrial sediments^4^. Organic-particle phytoclasts are lighter than equivalently sized inorganic silt (specific gravity, < 2.5 g/cm^3^ vs. 2.62–2.68 g/cm^3^) and can float for periods of more than 1 year^1,5,6^. Thus, organic material that moves along with terrestrial sediments can float for longer periods and migrate over longer distances than inorganic particles, transported by the current into the open ocean. Kawahata et al.^7^ reported that pollen grains originating from the East China Sea and the eastern part of the Japanese archipelago are transported by the Kuroshio Current into the Hess rise of the northwestern Pacific. In general, small phytoclasts (< 30 and ~ 35 µm) are deposited 10 and 20–35 km, respectively, from the coastline on the continental shelf adjacent to the open ocean, although the distance can vary depending on the depositional environment^8,9^. Hence, palynofacies assemblages of hemipelagic sediments consist mostly of marine origin palynomorphs, whereas phytoclasts are rare or absent^1,4^.

**Figure 1:**
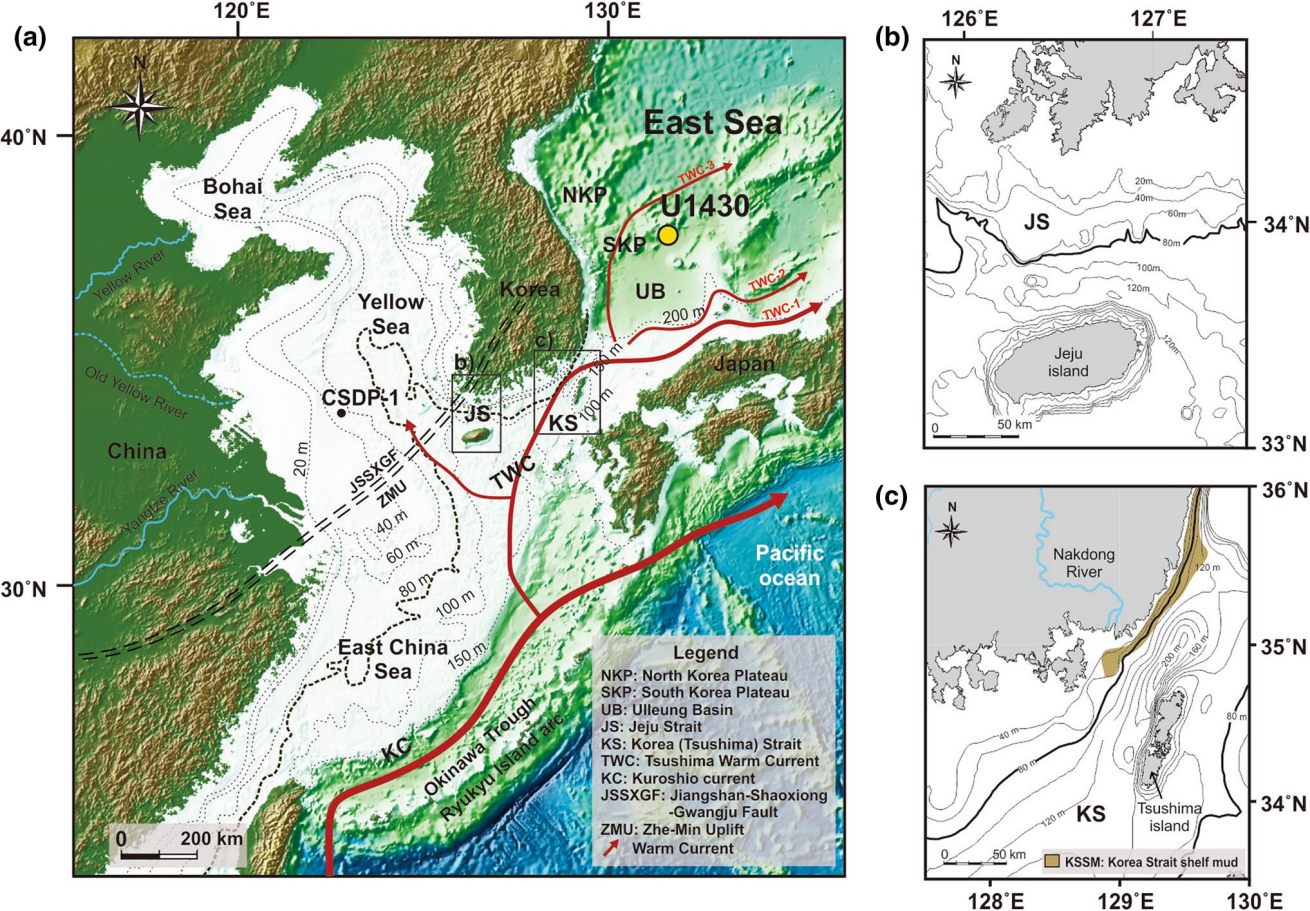
Physiographic maps of the study area. (**a**) Map showing the U1430 core site, bathymetry, current system, faults, and uplift in the East Sea, Yellow Sea, East China Sea, and adjacent regions (modified from Tada et al.^26^ and Liu et al.^48^. (**b**) Bathymetry map of the Jeju Strait region. (**c**) Bathymetry map of the Korea (Tshushima) Strait region^23^. These maps were created using the software Generic Mapping Tools (https://www.generic-mapping-tools.org/download/)^56^.

The study site, IODP Expedition 346 site U1430, is located at a water depth of 1072 m in the ESKP, which is in the northeastern part of the Ulleung Basin (UB) in the East Sea, 200 km from the eastern coast of the Korean Peninsula (KP; Fig. 1a). The East Sea, which is influenced by the East Asian Monsoon, is a semi-enclosed marginal sea connected via four straits to the Pacific Ocean and the East China Sea^10,11^. Sediment of various provenances enters the East Sea via monsoons and the Tsushima Warm Current (TWC) flowing through the Korea (Tsushima) Strait (KS). Most terrestrial sediments transported by currents into the East Sea originate from the Yangtze River and the western and southern sea shelves of the KP and flow into the East Sea through the Jeju Strait (JS) and KS^12–18^. The continental shelf of the South Yellow Sea [SYS; less than about 100 m below the sea floor (mbsf)] is composed of paleochannels and river mouths, including those of the Yangtze River^19,20^. The southern sea shelf of the KP is composed of a narrow shelf less than 100 m deep connected to the Seomjin and Nakdong river mouths. These continental shelves were completely exposed during the Last Glacial Maximum (LGM) when the eustatic sea level^21^ fell by a maximum of 140 m, and they alternated repeatedly between terrestrial and marine environments in response to eustatic sea-level changes during the Late Quaternary. The JS (less than about 160 m) is located between the southern coast of the KP and Jeju Island, which extends from the mouth of the Yangtze River to the southern sea of Korea (Fig. 1b). The KS is the main passage connecting the Yellow Sea, the Okinawa Trough, and the East Sea, and it has a maximum depth of 230 m (Fig. 1c). These straits maintained narrow paleochannel features during the LGM and thus played an important role in transporting terrestrial sediments from the Yellow Sea, the southern sea shelf of the KP, and other sites of origin^10,14,17,20,22,23^. As the water depths of the strait and shelves were included in the ranges of eustatic sea-level curves, the areas of exposed shelves and the water depths of main straits changed in response to eustatic sea-level changes; therefore, the distance from nearby sediment sources to the ESKP also changed. That is, in the East Sea, sea-level changes govern the distance from the sediment source.

The ESKP is an ideal study area in which to investigate marine environmental changes based on sediment characteristics and organic matter supplies in response to fluctuations in sea level. The reason for this is that the ESKP is influenced by the third branch of the TWC, which carries terrestrial sediments from nearby continental shelves and flows through the KS into the East Sea^24,25^ (Fig. 1a). We attempted to interpret the paleo-ocean environments of the ESKP using a known palynofacies model^8^. However, the model proved to be inapplicable in this study; it had been applied primarily to coastal and shelf areas adjacent to open ocean environments. Here, we address the new relationship between palynofacies assemblages and sea-level changes that occurred as a result of glacial–interglacial cycles during the last 2 Ma in the East Sea, a semi-enclosed ocean environment. Furthermore, the various marine environments and tectonic conditions that influence palynofacies characteristics of sediment flowing into the ESKP are discussed.

## Results

The U1430 continuous sediment core of almost 258 m was obtained from the ESKP by the IODP Expedition 346 project; the present study focuses on the top 56 m of this core. The study section is mainly composed of silty clay, clayey silt, and biogenic ooze. The detailed lithology was adopted from shipboard core descriptions^26^ (Supplementary Figure S1). The study section is divided into two units (Units IA and IB) at 46 m based on sedimentary features and color reflectance^26^. The sedimentary feature is characterized by alternating interbedded dark and light color sediments at a decimeter scale. This color alternation is clearly identified in the uppermost part of Unit IA and gradually decreases in frequency in Unit IB. The core lamination of alternating dark and light color bands is markedly observed in the Quaternary sediments of other cores of Expedition 346 as well as U1430 in the East Sea^27^. Tada et al.^27^ constructed the age models of each core by correlating the dark layers of U1422, U1423, U1424, U1425, U1426, and U1430. We adopted this age model for U1430 in this study (Supplementary Figure S2 and Supplementary Table S1).

Palynofacies analyses showed that the proportions of phytoclasts, palynomorph, and amorphous organic matter were 50%, 37.1%, and 12.9%, respectively (Supplementary Figure S3). The total concentration of phytoclasts (Tp) was 5991–84,430 (n/g), within which the concentration of coarse-grained phytoclasts (Cp) was 211–34,114 (n/g), and the concentration of fine-grained phytoclasts (Fp) was 4616–53,828 (n/g; Supplementary Figure S4). Tp was relatively constant, except in the uppermost 30 m section, where Cp increased and Fp decreased upward, respectively. In addition, Tp, Cp, and Fp exhibited clear relative fluctuations in the upper section above 30 m.

To confirm the glacial period in question in this study, we identified cold-arid climate indicator species, such as *Picea*, *Abies*, *Pinus-Haploxylon*, and *Artemisia*. The conifers *Picea*, *Abies*, and *Pinus-Haploxylon* inhabit the subalpine zone inland of northern East Asia, tolerate cold and arid climate conditions, and are well-known indicators of cold-dry climate conditions^28–32^. Moreover, the xerophytic herb pollen of *Artemisia* inhabits the open grasslands inland under cold and arid climate conditions and is used as an indicator of arid climate conditions^33,34^. The pollen results indicated that coniferous tree pollen dominated throughout the entire section and showed likely periodic fluctuations, whereas *Artemisia* was rare in the lower part and increased upward (Supplementary Figure S5).

## Discussion

**Figure 2:**
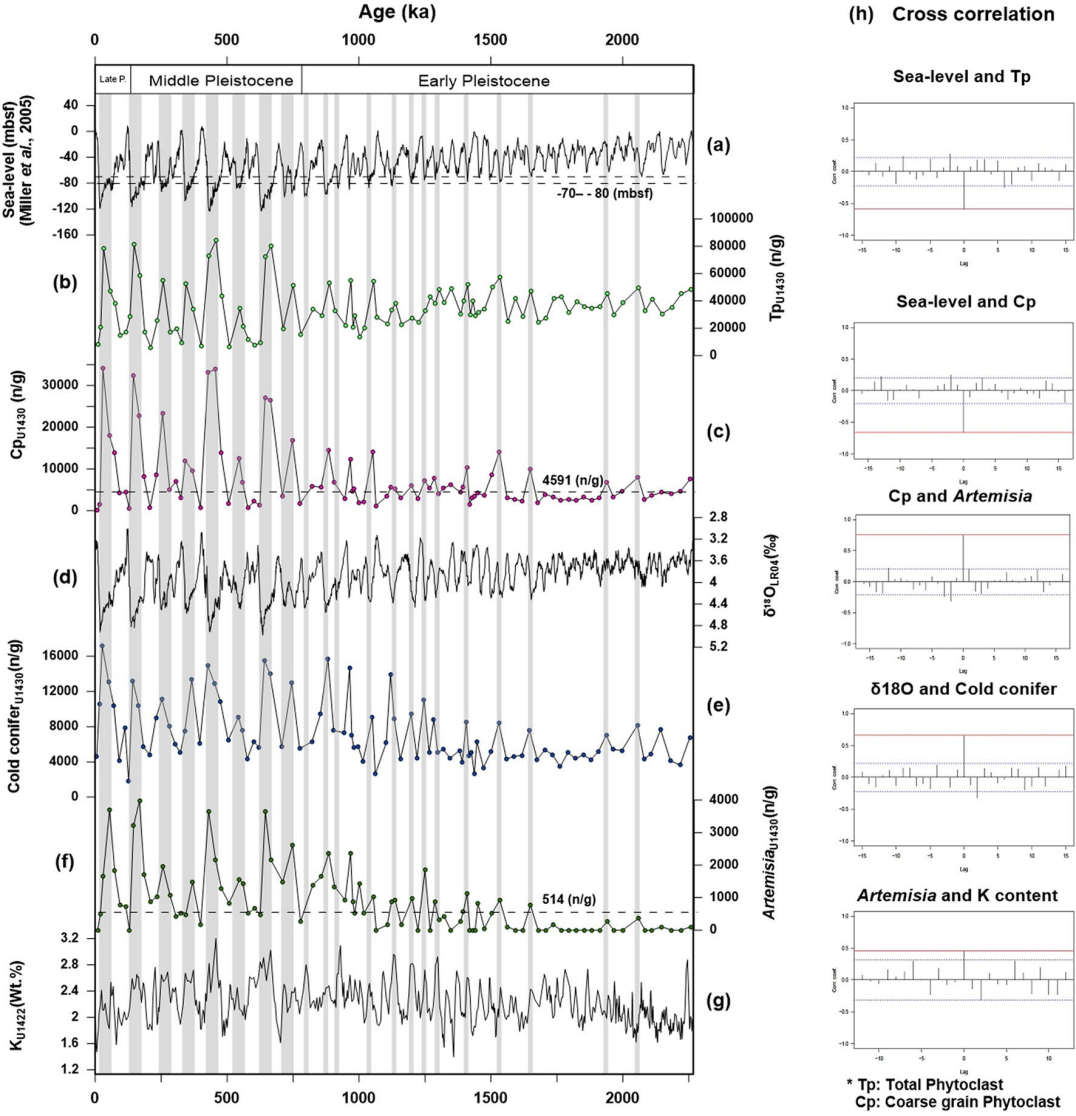
Multi-index comparison of phytoclast, pollen, sea-level, δ^18^O, and potassium records during the 2 Ma. (**a**) Global (eustatic) sea-level records^21^. (**b**) Total concentration of phytoclasts (Tp; n/g). (**c**) Concentration of coarse-grained phytoclasts (Cp; n/g): lath shaped (length:width ratio at least 3.0), larger than 80 µm. (**d**) LR04 stacks of benthic foraminiferal δ^18^O values^38^. (**e**) Pollen concentrations of cold climate indicators from Northern East Asia; sum of *Abies*, *Picea*, and *Pinus-Haploxylon*^28–32^. (**f**) Concentration of xerophytic herb pollen of *Artemisia*^33,34^. (**g**) Potassium (K) content (wt.%) quantified from natural gamma radiation (NGR) spectra of the site U1422 at the East Sea, indicating arid climate conditions^36,43^. (**h**) Cross-correlation of eustatic sea level^21^, Tp, Cp, cold-dry indices pollen, δ^18^O value^38^, and K content. Tp and Cp are both negatively correlated with sea level. Positive correlations with no lag time were observed between Cp and *Artemisia*, between δ^18^O values^38^ and cold conifer, and between *Artemisia* and K (wt.%) content^37^. The gray bars represent periods of low sea level characterized by high Tp, Cp, δ^18^O, cold-conifer, *Artemisia*, and K content values. This diagram is created using Grapher-12 software (https://www.goldensoftware.com).

In general, marine palynomorphs are more prevalent than phytoclasts in hemipelagic sediments^1,4^. However, despite the presence of hemipelagic sediments, the entire section of the U1430 core is dominated by phytoclasts (average: 50%)—represented by coarse-grained phytoclasts larger than 80 µm and lath shaped—rather than palynomorphs (average: 37.1%; Supplementary Figures S2 and S3). Palynofacies analyses indicate that the overall pattern of phytoclasts is negatively correlated with the eustatic sea-level curve (Fig. 2a,b,h), which suggests that terrestrial phytoclasts were discharged from the nearby exposed continental shelves at the lower sea-level stage during the glacial period. This result is at odds with the high sediment discharge at the continental shelves transported by heavy precipitation at the high sea-level stage during the interglacial period. According to pollen records from the same section, conifer tree and herb pollen grains are used as indicators of cold-arid climate, such as those from *Abies*, *Picea*, *Pinus-Haploxylon*, and *Artemisia*, are dominant when total phytoclasts are abundant (Fig. 2b,e,f). Potassium is a component of illite, which is generally formed under cold and dry climate conditions; hence, the high K (wt.%) content indicates an enhanced arid climate condition^35^. Shipboard-measured natural gamma radiation (NGR) data from the IODP Expedition 346 may be used to estimate the K, U and Th content, and the K content quantified from NGR spectra may be used to estimate dust input variability and aridity without clay mineral analysis^36^. Zhang et al.^37^ reported that the K (wt.%) content quantified from NGR spectra of site U1422 at the East Sea correlates positively with the δ^18^O value of the LR04 stack^38^ and suggests the aridity of central Asia. Based on the palynomorph records, the cold-conifer and *Artemisia* pollen concentrations indicate a positive correlation with the δ^18^O value of the LR04 stack^38^ and K (wt.%) content of U1422^37^ (Fig. 2d–h). These findings indicate that conifers inhabited China and the KP under cold and arid climate conditions during cold periods, such as the LGM, and that cold-climatic conifer pollen and eolian dust were transported by the East Asian winter monsoon. Meanwhile, as sea levels fell, the exposed continental shelves of the SYS and southern sea of the KP were covered by xerophytic herb, *Artemisia*, which would have been delivered into the East Sea by the TWC. The period showing high concentrations of phytoclasts clearly indicates the cold-arid climate conditions of the glacial period. We suggest therefore that the main factor controlling the influx of phytoclasts into the ESKP is the change in distance from the source area due to the fall in eustatic sea level^21^ during the glacial period, rather than rainfall intensity during the interglacial period.

Cp, wood-derived plant fragments larger than 80 µm and lath shaped, is usually observed in the palynofacies of coastal and terrestrial sediments near the plant source area. However, as noted above, Cp is common in the entire section of the U1430 core, although it is contained in hemipelagic sediment. Moreover, it was high (above 4591 n/g) during the glacial period, when sea levels fell by more than 70–80 mbsf (Fig. 2a,c). According to the eustatic sea-level curve during this period, the shelves would have been completely (maximally) exposed from the SYS to the southern sea of the KP. Hence, we may consider that Cp originated from the southern sea shelf of the KP that was closer to the ESKP. Cp is also positively correlated with *Artemisia* data used as an indicator of cold-dry climate and is transported by water. Therefore, we conjecture that Cp deposited in exposed shelves along the southern sea shelf of the KP during the glacial period was first discharged by freshwater to the paleochannel and then transported by marine currents into the East Sea.

**Figure 3:**
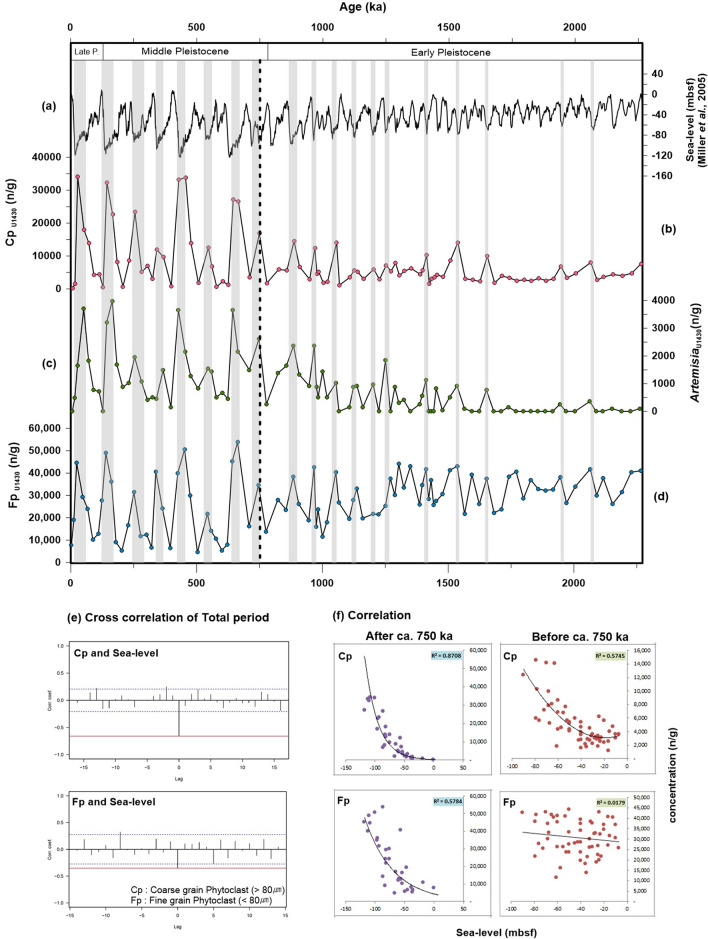
Correlations between concentration of coarse-grained phytoclasts (Cp), concentration of fine-grained phytoclasts (Fp), and global sea-level data. (**a**) Global (eustatic) sea-level records^21^. (**b**) Concentration of coarse-grained phytoclasts (Cp; n/g): lath shaped (length:width ratio at least 3.0), larger than 80 µm. (**c**) Concentration of xerophytic herb pollen *Artemisia*^30,31^. (**d**) Concentration of fine-grained phytoclasts (Fp; n/g): smaller than 80 µm. (**e**) Cross-correlations among Cp, Fp, and sea level during the period ca. 2 Ma. Cp and Fp are both negatively correlated with sea level with no lag time. (**f**) Correlation coefficients of Cp and Fp each compared to sea level, which clearly increased at ca. 750 ka. The gray bars represent low sea-level periods characterized by high Cp, Fp, and *Artemisia* concentrations. Dashed vertical black lines denote the timing of increases in the amplitude of the sea-level curve and Cp, *Artemisia*, and Fp values. This diagram is created using Grapher-12 software (https://www.goldensoftware.com).

Like Cp, Fp is negatively correlated with eustatic sea-level curves. However, the correlation coefficient is lower than that of Cp (Fig. 3a–e). This indicates that Fp may be less affected than Cp by fluctuations in sea level; it is likely that the less correlated Fp originated farther away than Cp. Fine-grained sediments of Korea Strait shelf mud (KSSM) have also been reported to originate from distant sources, such as the SYS, despite being heavier than Fp^14,17,23^. Therefore, we believe that Fp originated not only from the southern sea shelf of the KP but also from the SYS, and it is likely that it flowed through the JS and KS into the East Sea.

Based on concentrations of phytoclasts over the past 2 Ma, Cp and Fp each showed different correlation ratios at ca. 750 ka (Fig. 3f). As the gray bars shown in Fig. 3b, Cp concentrations show above 10,000 n/g when the sea-level fell by more than 70–80 mbsf. Before ca. 750 ka, the Cp concentration rarely exceeded 10,000 n/g because the sea level did not decrease by more than 70–80 mbsf during the warm period. Therefore, the Cp concentration showed a moderate correlation (R^2^ = 0.57; the moderate concentration at low sea levels; Fig. 3f). However, after ca. 750 ka, was highly correlated (R^2^ = 0.87; the high concentration at low sea levels; Fig. 3f). These results may be correlated with an increase in the amplitude of sea-level changes due to climatic fluctuation shifting from 41 to 100 ky periodicity after the Mid-Pleistocene Transition (MPT)^39–41^. Furthermore, *Artemisia* showed a gradual increasing trend prior to ca. 750 ka, which is most evident between 750 and 1500 ka (Fig. 3c and Supplementary Figure S6). This gradual increase is concordant with gradual global aridification and cooling after the MPT.

**Figure 4:**
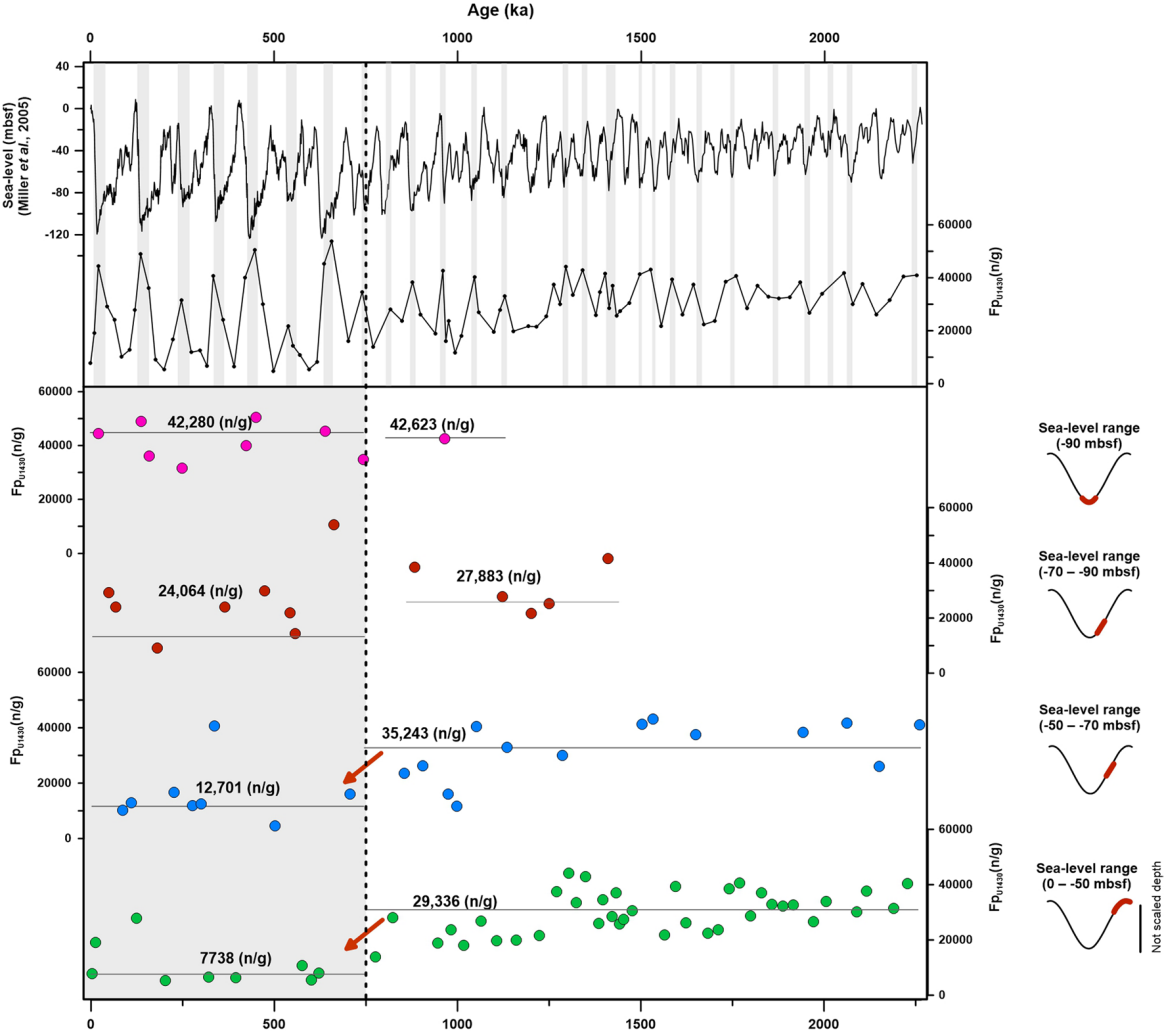
Correlations between concentration of fine-grained phytoclasts (Fp) and certain sea levels during the ca. 2 Ma. Fp concentrations were high (median, 27,883–42,623 n/g) when the sea level fell by more than 70 mbsf (i.e., in the proximate source area, according to the modern water depth line). When the sea level fell by less than 50 mbsf (i.e., in the distant source area, according to the modern water depth line), Fp concentrations remained high (29,336–35,243 n/g) before ca. 750 ka, but decreased (to 7738–12,701 n/g) after ca. 750 ka. The gray bars in the upper section denote anti-phase correlations between the sea level and Fp concentration. The gray area in the left lower section indicates Fp concentrations after ca. 750 ka. This diagram is created using Grapher-12 software (https://www.goldensoftware.com).

**Figure 5:**
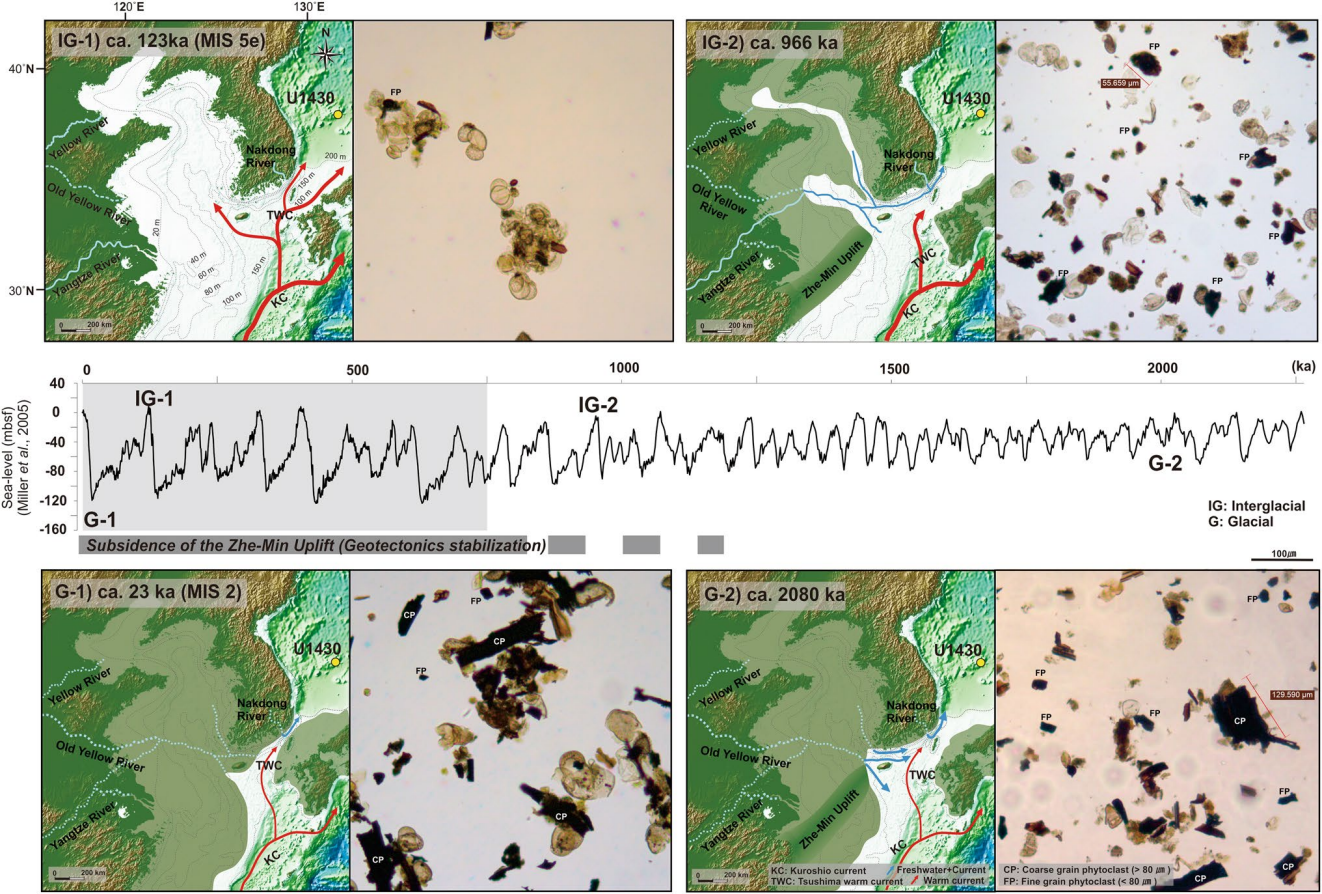
Schematic reconstructions of paleoceanography at interglacial (IG-1 and IG-2) and glacial (G-1 and G-2) periods inferred from changes in the palynofacies during the ca. 2 Ma. Prior to ca. 750 ka, the palynofacies did not clearly change between the glacial and interglacial periods. The SYS was high terrain, and the sea-level curve had a low amplitude. (G-2) ca. 2080 ka, glacial period: Fp was dominant and Cp was common. Cp and Fp were supplied by the freshwater discharged from exposed nearby shelves and the weak TWC. Exposed southern sea shelves of the KP, which supplied Cp, were narrow because the sea level had fallen by only a maximum of 70 m during this period. (IG-2) ca. 996 ka, interglacial period: Fp was dominant and Cp was rare. Despite the high stand sea-level period, the SYS maintained a high terrain; considerable amounts of Fp could have been supplied by the freshwater from the Yangtze River and other rivers from the KP. After ca. 750, the palynofacies clearly changed between the glacial and interglacial periods. Depending on the subsidence of the ZMU, the geotectonics of the East China Sea area were stabilized and formed the modern-like coastline. Furthermore, the sea-level curve had high amplitude. (G-1) ca. 23 ka, LGM: Cp was dominant. The global sea level^21^ fell by 140 m, the southern sea shelves of the KP were maximally exposed, and much Cp was transported by the freshwater and the weak TWC. (IG-1) ca. 123 ka, MIS 5e: Fp was dominant. The modern-like coastline was formed during the high stand sea-level period, and the source area became further away; only Fp was transported over long distances by the TWC. This illustration is created using CorelDRAW Graphics Suite X7 software (https://www.coreldraw.com).

Fp also shows a general decreasing trend between 750 and 1500 ka (Fig. 3d), but this trend correlates only weakly with the sea level (R^2^ = 0.02; Fig. 3f); no significant difference in the correlation coefficient was observed around 1500 ka (Supplementary Figure S6). Before ca. 750 ka, Fp fluctuated more than Cp did due to the high concentration, but this does not coincide completely with the glacial–interglacial cycles. As Fig. 3f shows, before ca. 750 ka, the Fp concentration was not related to sea-level changes. This result suggests that Fp was affected by a factor other than sea-level changes at that time. During the past 2 Ma, Fp concentrations remained high (24,064–42,623 n/g) when the sea level fell by more than 70 mbsf (i.e., in the proximate source area, according to the modern water depth line; Fig. 4). In the distant source area, where the sea level fell by less than 50 mbsf, Fp concentrations remained high (29,336–35,243 n/g) before ca. 750 ka, but decreased (7738–12,701 n/g) thereafter (Fig. 4). Prior to ca. 750 ka, Fp concentrations remained high even when the sea level fell by less than 50 mbsf (i.e., in the distant source area), suggesting that Fp continued to supply the ESKP before ca. 750 ka, regardless of the drop in sea level. Hence, we considered that other factors, such as global climate conditions and geotectonic events, affected Fp before ca. 750 ka. First, Fp might have been transported by sediment discharge from the nearby continent due to precipitation prior to ca. 750 ka, when the global climate was mild. Our finding that concentrations of cold-conifer and *Artemisia* pollen were low prior to ca. 750 ka (Fig. 2) are in line with previous reports indicating that the East Asian climate was warmer and more humid prior to the MPT^39–42^. Sun et al.^42^ reported that reconstructed mean annual precipitation values for the last 2 Ma correlated positively with the δ^18^O value of the LR04 stack. According to this result, Fp concentrations should be much higher during the interglacial period due to large amounts of precipitation; our results, however, show that Fp concentrations were higher during the glacial period, when less precipitation occurred (Fig. 4). Hence, we considered that factors other than climate conditions, such as geotectonic events, affected Fp before ca. 750 ka. The Himalaya–Tibetan Plateau (HTP) gradually uplifted after the Indo-Eurasia plate collision ca. 50–60 Ma, causing the Yellow Sea Basin to maintain its continental environments up to the Pleistocene^43,44^. Fluvial deposits from several cores, including CSDP-1 on the Yellow Sea Basin until the late Pleistocene, also suggest that the SYS included continental/fluvial environments up to this period^19,44–47^. Moreover, the Zhe-Min uplift (ZMU), which extends from southern China to the western part of the Jeju Strait along the Jingshan–Shaoxing–Gwangju Fault, and was high terrain until the late Pleistocene, played the role of a partial barrier, preventing the intrusion of seawater from the East China Sea^44,46^. The HTP uplift occurred more rapidly around the MPT, and after 0.8 Ma, the SYS and ZMU may have subsided as a result of isostatic balance with the HTP and Ryukyu Island arc, forming the modern-like coastline^44,46–49^. Hence, we assume that the maintenance of high Fp concentrations prior to ca. 750 ka, even as the sea level fell by less than 50 mbsf, required a continuous supply of Fp from the SYS, which exposed the lowland regardless of sea-level fluctuations due to the high terrain of the ZMU. After ca. 750 ka, as before, Fp concentrations were low even when the sea level fell less than 50 mbsf, indicating that the Fp source gradually moved further away from the ESKP because of the formation of a modern-like coastline following the subsidence of the SYS and ZMU (Figs. 4, 5). However, as noted above, Cp may originate from the southern sea shelf of the KP, which was closer to the ESKP. Therefore, we assumed that the Cp concentration has not been affected much by geotectonic events. In conclusion, the changes in phytoclast facies controlled by both eustatic sea-level changes in the glacial–interglacial cycle and the geotectonic event in the early to mid-Pleistocene are peculiar characteristics of the semi-enclosed East Sea, including the narrow straits through which the terrestrial sediment entered (Fig. 5). The overall pattern in the total concentration of phytoclasts shows a negative correlation with the eustatic sea-level curve and is higher after the subsidence of the ZMU.

Prior to ca. 750 ka, changes in phytoclast facies in response to sea-level changes (glacial–interglacial cycle) were not clear because large amounts of Fp originating from the SYS and the high terrain of the ZMU were transported into the ESKP regardless of sea-level changes. By contrast, after ca. 750 ka, the modern-like coastline was formed as the ZMU subsided and began to be influenced by the global sea-level changes in the 100 ka cycle; thus, changes in the palynofacies were clearly observed according to eustatic sea-level changes (the glacial–interglacial cycle; Fig. 5 and Supplementary Figure S7). In particular, Fp of the distant source (the SYS and beyond), containing MIS 5e, occurred during the interglacial period, whereas Cp of the near source (the southern shelf of the KP), such as MIS 2 (LGM), dominated during the glacial period (Supplementary Figure S7). This indicates that phytoclasts mainly flowed into the East Sea during the glacial period, when the sea level fell, rather than during the interglacial period, which was characterized by heavy precipitation. Our study suggests that concentrations of phytoclasts were governed by changes in the water depths of straits and shelves due to changes in sea level. We expect that this newly observed palynofacies characteristic may be attributable to the semi-enclosed ocean environment connected by narrow straits.

## Methods

For palynofacies analyses, a total of 91 dry samples were processed following a standard palynological pretreatment method^3^. Two exotic *Lycopodium* tablets were added to each sample to calculate organic particle concentrations^50^. For the palynofacies analyses, 500 organic particles were counted per slide until at least 250 *Lycopodium* spores were counted^4^. The identified organic particles were classified into three groups: phytoclasts (two types according to size and shape), palynomorphs (marine and terrestrial origin), and amorphous organic matter (marine origin AOM). In particular, phytoclasts varying according to size, shape, color (translucency), and the presence or absence of microstructure were observed in this study. Thus, we categorized phytoclasts composed of brown-black plant fragments into two groups based on the classification methods developed by Dybkjaer^51^ and Tyson and Follows^8^: Cp (coarse-grained phytoclasts) were larger than 80 µm and lath shaped (length:width ratio at least 3.0), and Fp (fine-grained phytoclasts) were 10–80 µm in size. Organic particles were identified with an optical microscope at 200 × and 400 ×, and pollen identification was conducted with reference to illustrations of pollen from northern East Asia^29,52,53^. Cross correlation was conducted with R 3.6.2^54^ using the BINCOR package^55^.

## Supplementary information

Supplementary Information.
