# Supplementary material for: New findings on palynofacies characteristics of semi-enclosed deep-sea environments in the East Sea over 2 million years
Source: Sci Rep. 2020 Oct 2;10:16432. doi: 10.1038/s41598-020-73493-3 (PMC7532222; doi:10.1038/s41598-020-73493-3)
Supplement: Supplementary file 1 — Supplementary Information. [file 41598_2020_73493_MOESM1_ESM.doc]

Supplementary Information for

**New findings on palynofacies characteristics of semi-enclosed deep-sea environments in the East Sea over 2 million years**

## Yongmi Kim^1, 2^, Sangheon Yi^1, 2*^, Chang-Pyo Jun^2^ , Eunmi Lee^2, 3^, Gil Young Kim^2^

^1^Korea University of Science and Technology, Daejeon 34113, Korea

^2^Korea Institute of Geoscience and Mineral Resources, Daejeon 34132, Korea

^3^Kangwon National University, Chuncheon 24341, Korea

*^*^*Correspondence to shyi@kigam.re.kr

**Supplementary figures**

1. **Figure S1.** Lithology of Units IA and IB at site U1430, with photographs of the study sections
2. **Figure S2.** Location map for the Expedition 346 core sites used to construct the age model
3. **Figure S3.** Cumulative percentages of the palynofacies group components examined in this study
4. **Figure S4.** Diagram of age-controlled concentrations of phytoclasts from this study
5. **Figure S5.** Diagram of age-controlled concentrations of cold-climate pollen indices from this study
6. **Figure S6.** Correlations among global sea-level data, *Artemisia* occurrence, and concentrations of fine-grained phytoclasts (Fp)
7. **Figure S7.** Photographs showing the palynofacies present between the glacial and interglacial periods

**Supplementary table**

1. **Table S1.** Estimated age of the U1430 core

**Supplementary reference list**

**Figure S1.** Lithology of Units IA and IB at site U1430, with photographs of the study sections (after Tada *et al*.^1^). Units IA and IB are divided at 46 m. Well-laminated Unit IA includes clearly observed color alternation and gradually decreases in alternate frequency in Unit IB. This photograph is edited using CorelDRAW Graphics Suite X7 software (www.coreldraw.com).

**
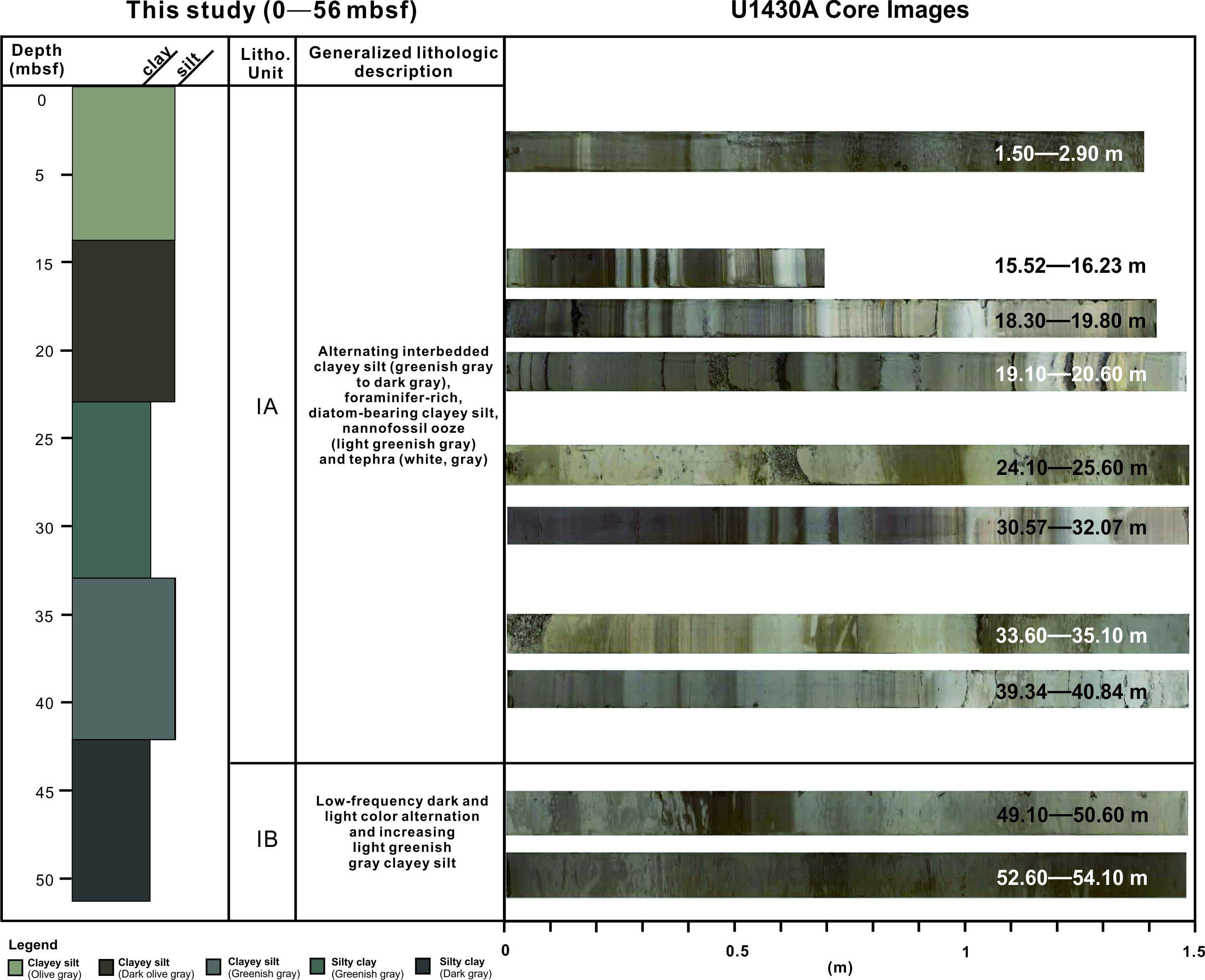
**

**Figure S2**. Location map for the Expedition 346 core sites used to construct the age model used by Tada *et al.*^2^. The map was drawn using the software Generic Mapping Tools (www.generic-mapping-tools.org/download/)^3^.


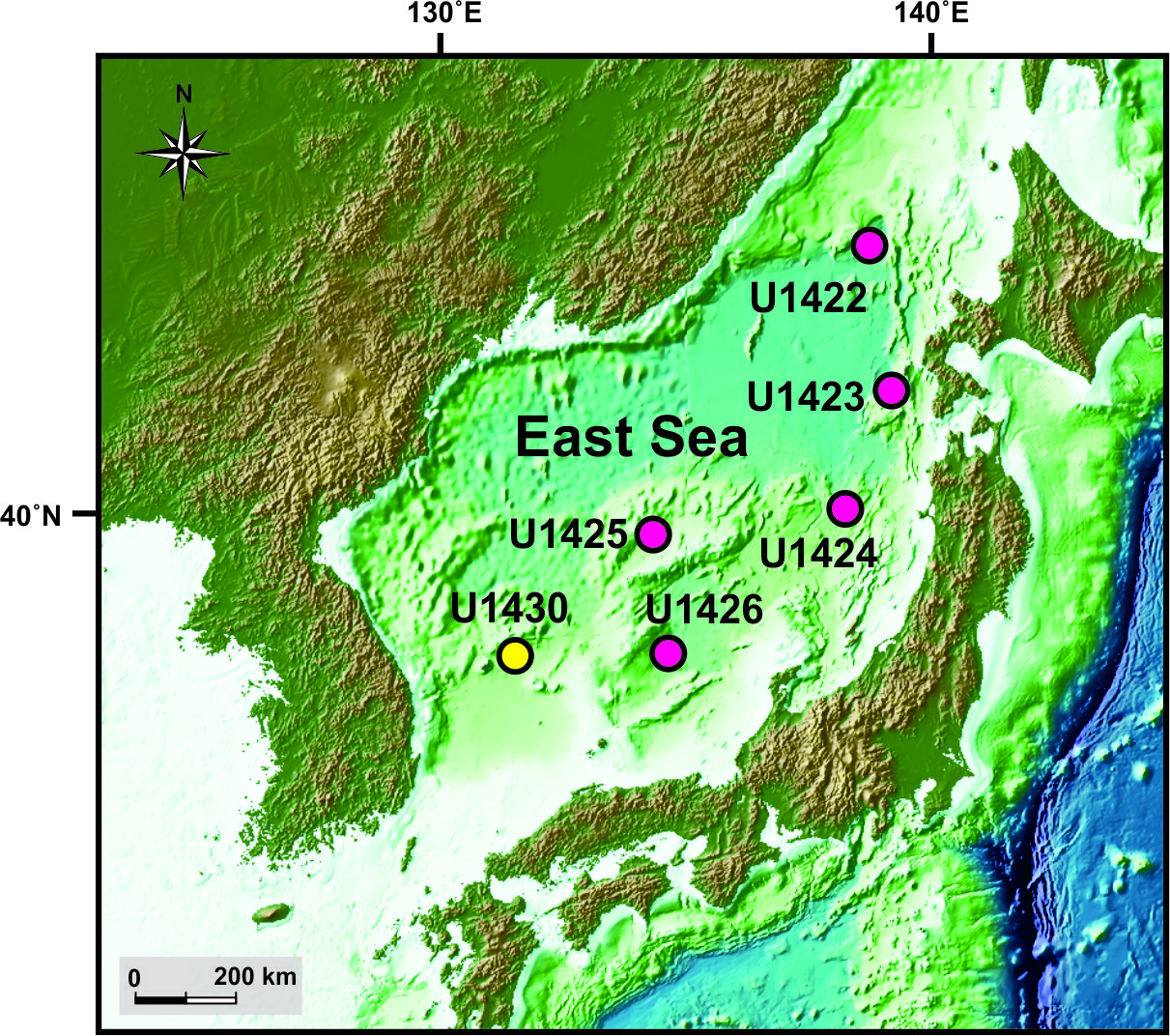


**Figure S3.** Cumulative percentages of the palynofacies group components examined in this study. The palynofacies group was classified into three groups: palynomorphs, phytoclasts, and amorphous organic matter (AOM). This diagram is edited using CorelDRAW Graphics Suite X7 software (www.coreldraw.com).


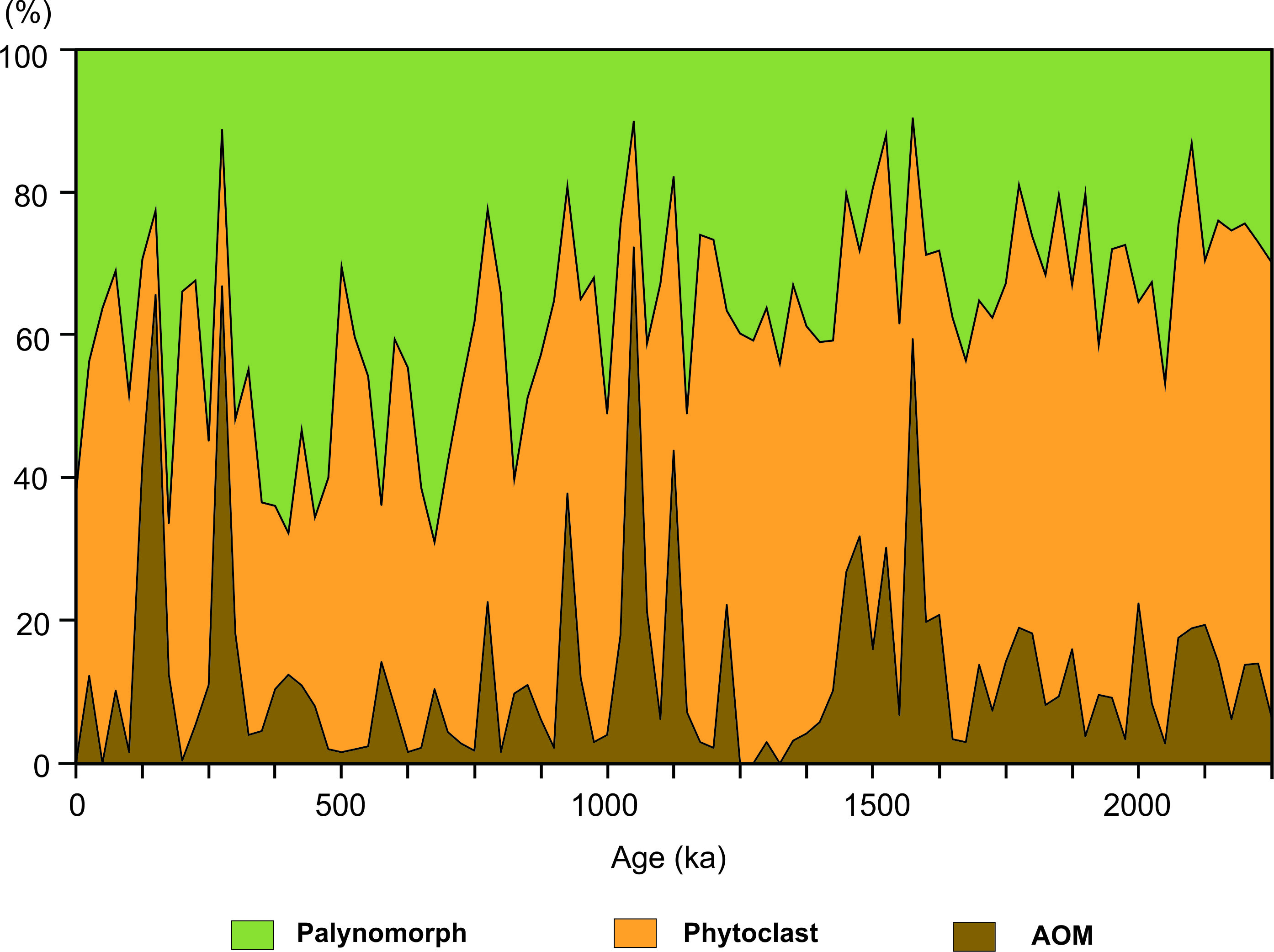


**Figure S4.** Diagram of age-controlled concentrations of phytoclasts from this study, created using the Tilia software.^4^


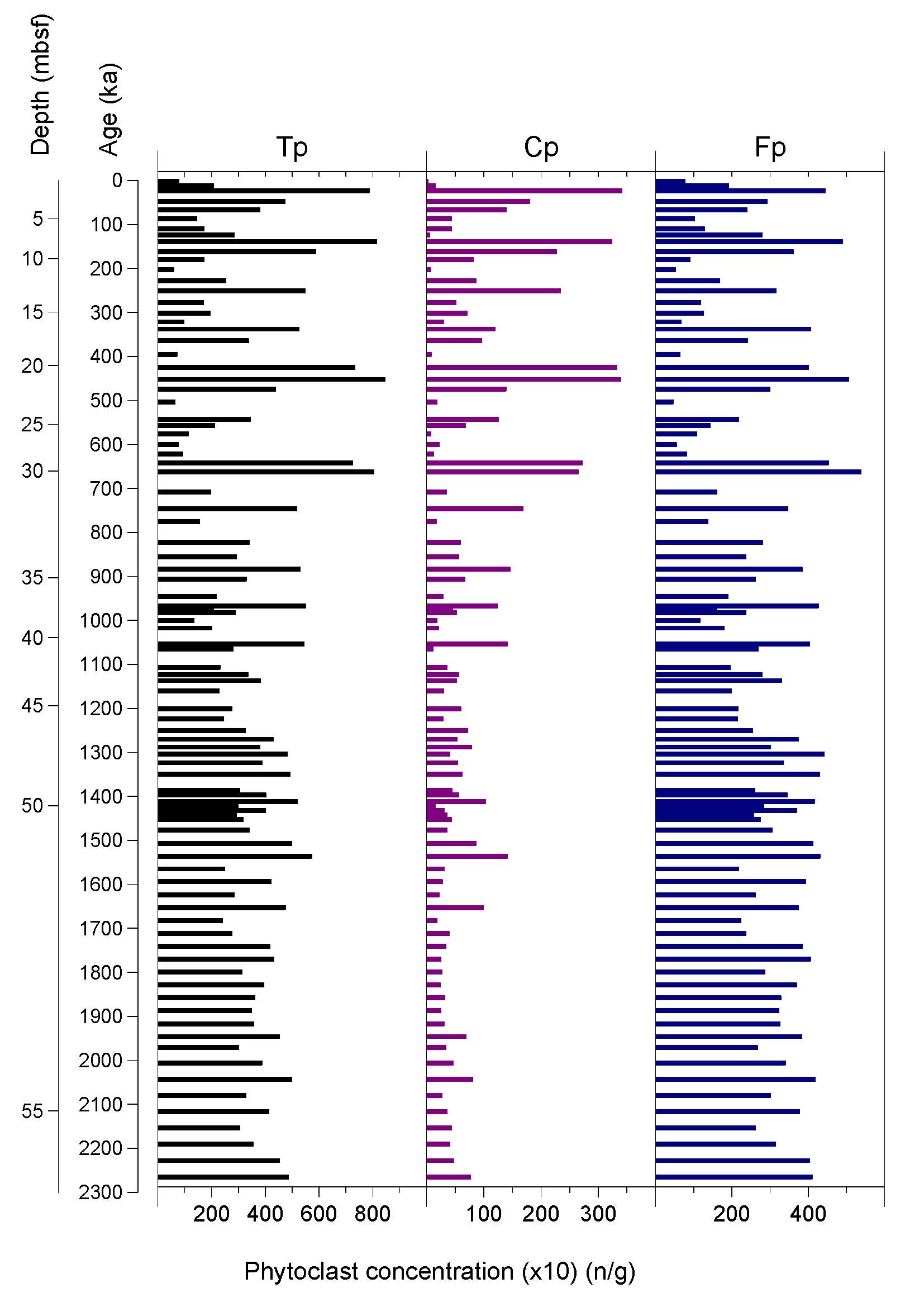


**Figure S5.** Diagram of age-controlled concentrations of cold-climate indices pollen from this study, created using the Tilia software.^4^


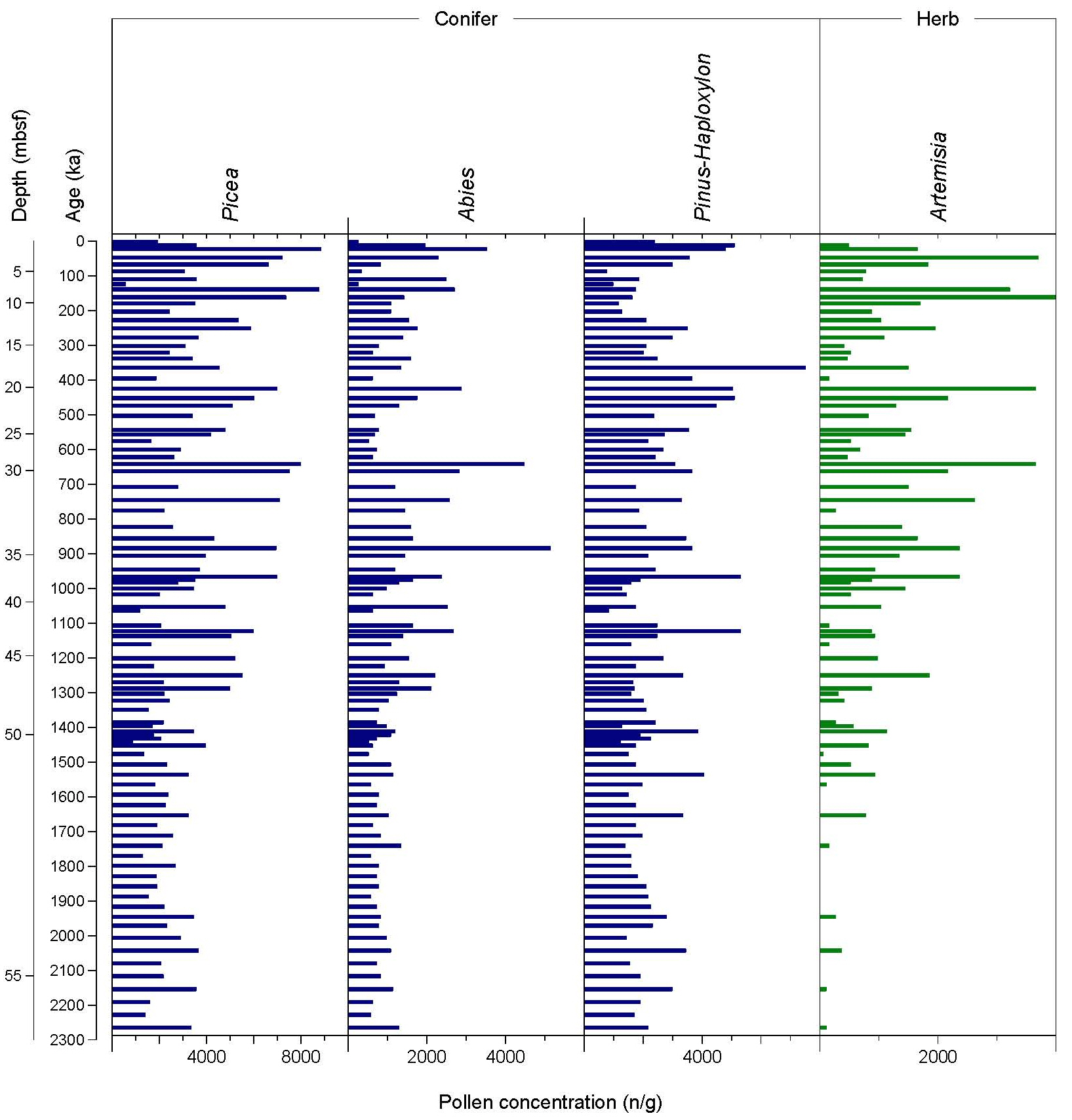


**Figure S6.** Correlations among global sea-level data, *Artemisia* occurrence, and concentrations of fine-grained phytoclasts (Fp). (a) Global (eustatic) sea-level records. (b) Concentrations of pollen from the xerophytic herb *Artemisia.* (c) Concentrations of Fp (<80 µm; n/g). (d), (f) Coefficients of correlation between *Artemisia* and Fp in relation to sea level, which clearly increased after ca. 750 ka. Prior to ca. 750 ka, correlation coefficients for *Artemisia* were normal, and values increased gradually. At ca. 1500 ka, coefficients for Fp are low, reflecting no clear correlation. This diagram is created using Grapher-12 software (www.goldensoftware.com)

.


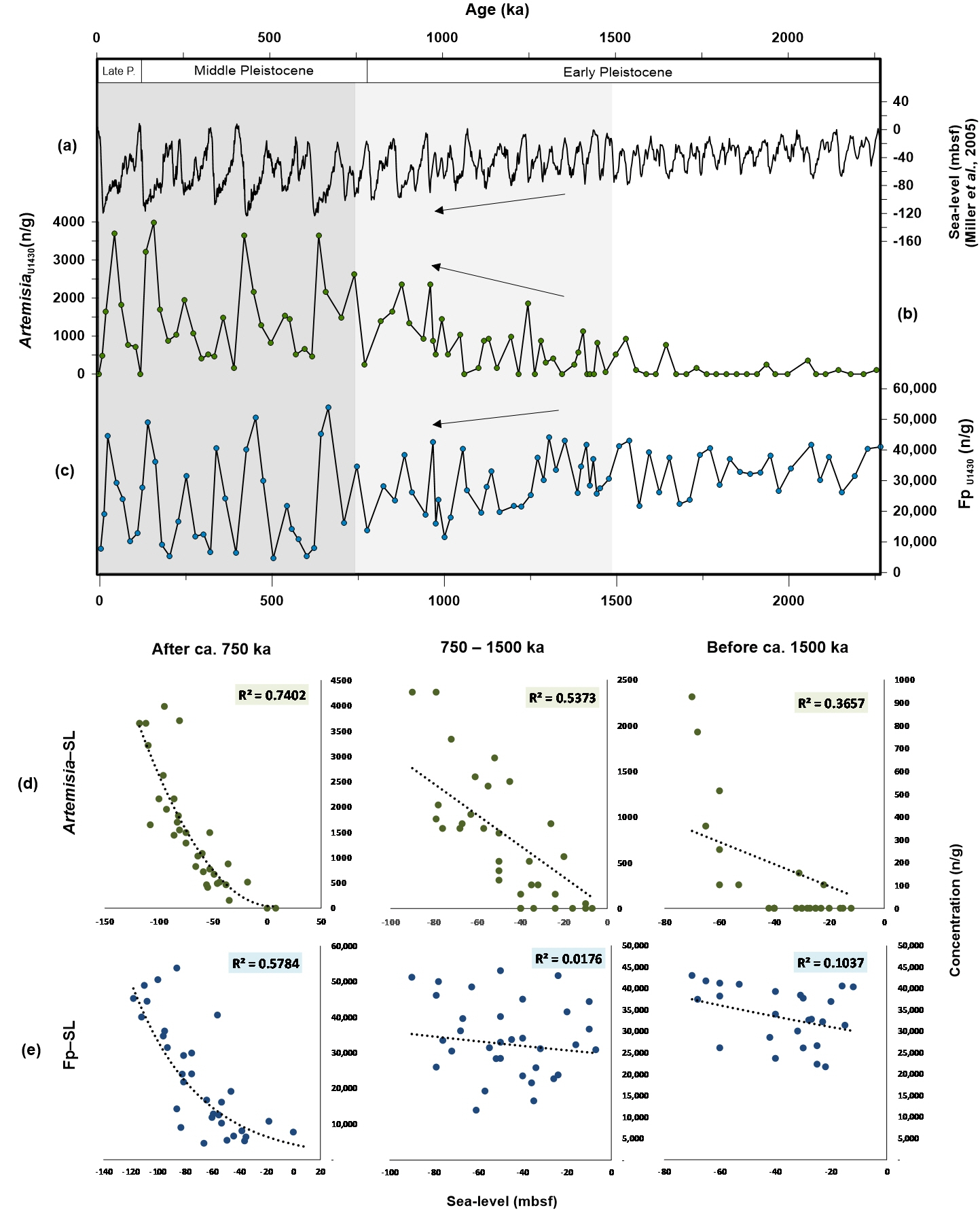


**Figure S7.** Photographs showing the palynofacies present between the glacial and interglacial periods. Before ca. 750 ka, changes in the palynofacies were not clearly distinguishable between the glacial and interglacial periods; after ca. 750 ka, changes in the palynofacies were apparent. This photograph is created by Leica microscope DM-1000 (www.leica-microsystems.com)


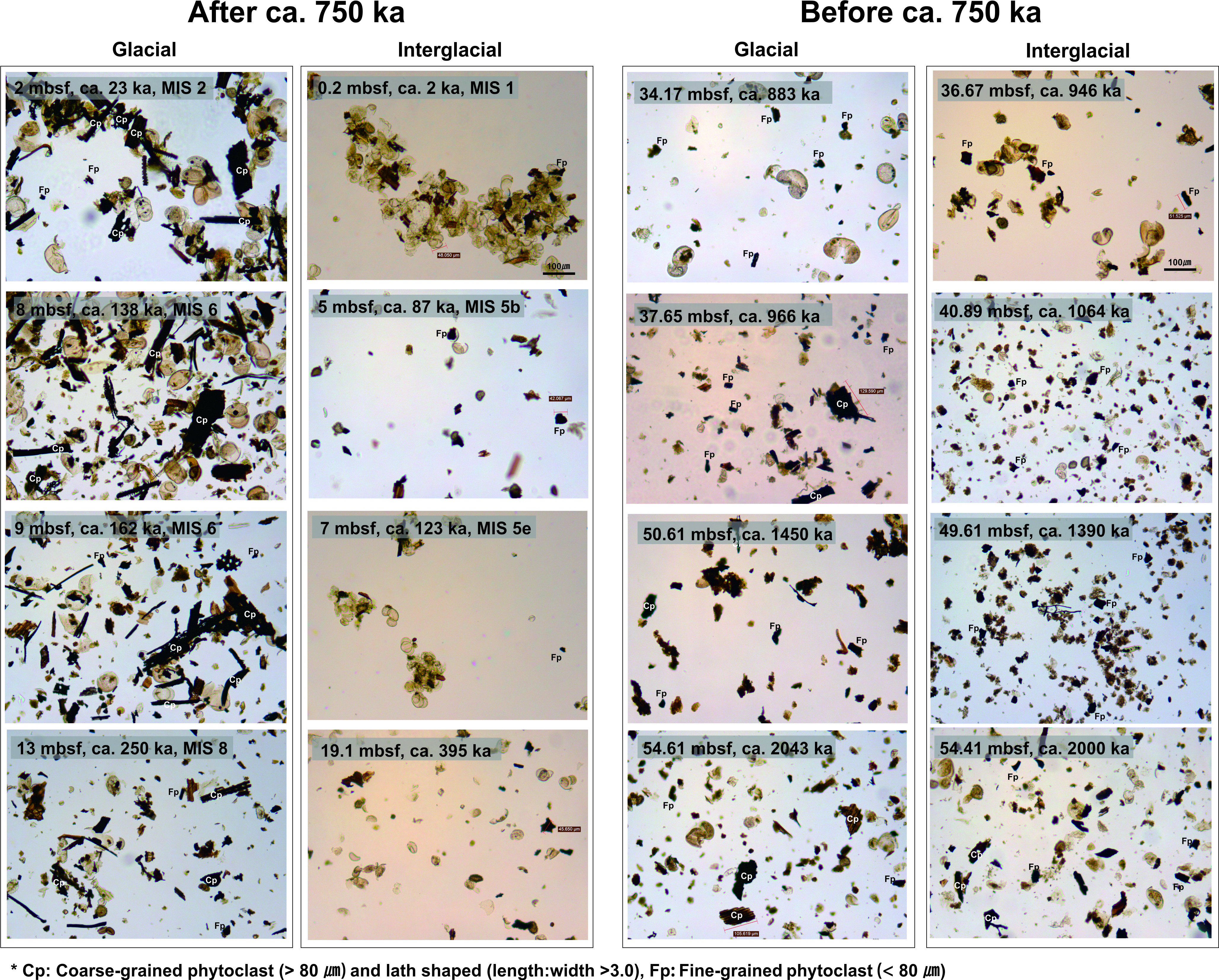


**Table S1** Estimated age of the U1430 core, based on comparison with the well-dated U1424 and U1430 cores (after Tada *et al.*^2^).


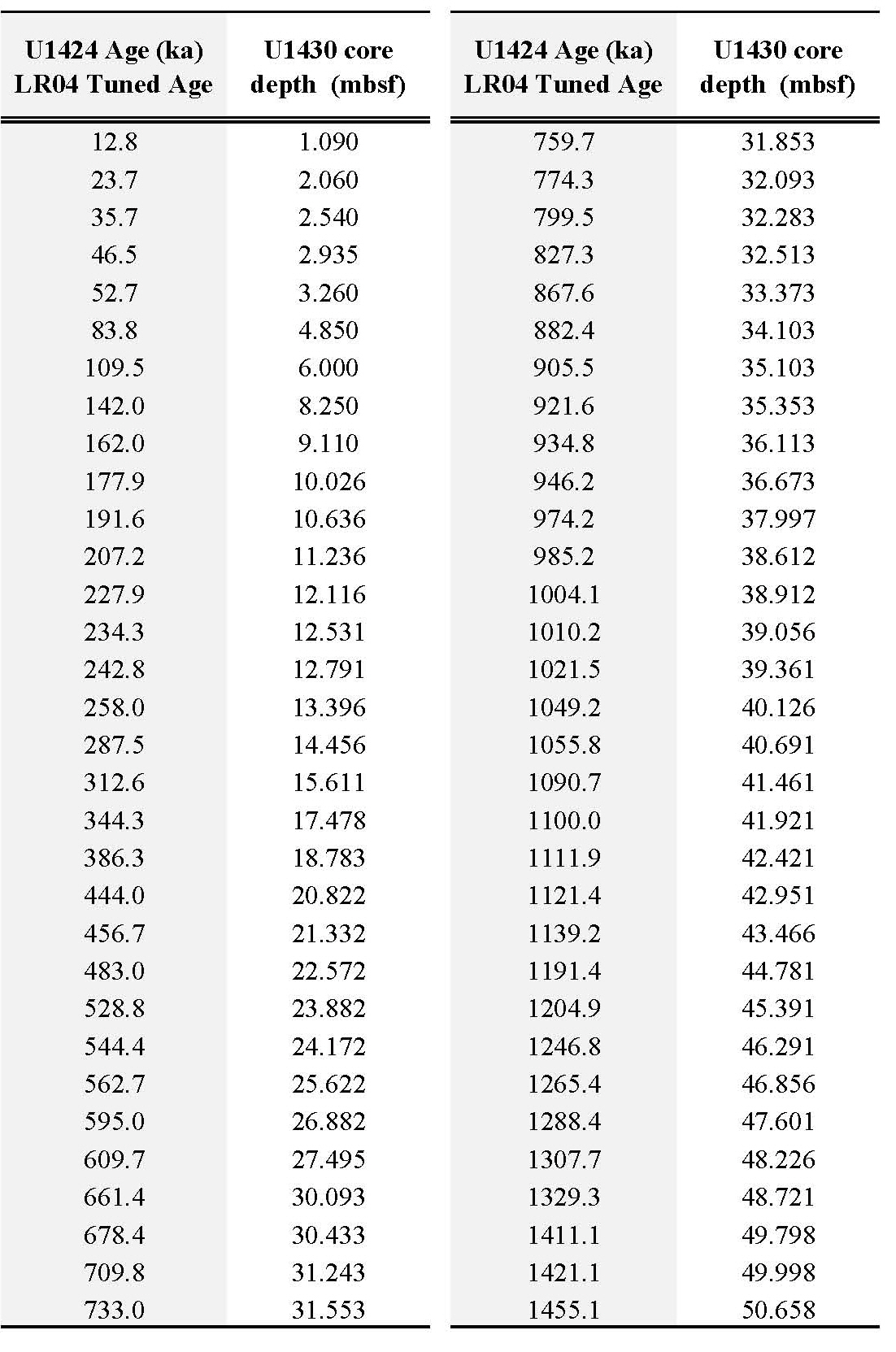


**References**

1. Tada, R., Murray, R.W., Alvarez Zarikian, C. A. and the E. 346 S. IODP Exp.346 Proceedings Site U1430. *Proc. Int. Ocean Discov. Progr.* **346**, 1–113 (2015).

2. Tada, R. *et al.* High-resolution and high-precision correlation of dark and light layers in the Quaternary hemipelagic sediments of the Japan Sea recovered during IODP Expedition 346. *Prog. Earth Planet. Sci.* **5**, 19 (2018).

3 Wessel, P., Luis, J. F., Uieda, L., Scharroo, R., Wobbe, F., Smith, W. H. F., & Tian, D. The Generic Mapping Tools version 6. *Geochemistry, Geophysics, Geosystems* **20**, 5556–5564 (2019).

4. Grimm, E. C. TILIA 1.7. 16. *Illinois State Museum. Res. Collect. Cent.* (2011).
